# Discovery of quality markers of *Meconopsis quintuplinervia* based on an absorption-based metabolite approach and rapid quantification using polarity-switching UPLC-QQQ-MS/MS

**DOI:** 10.3389/fphar.2024.1474768

**Published:** 2024-12-06

**Authors:** Yifan Tian, Yuan Liu, Yuanlin Kong, Limin Li, Zhengming Yang, Wenbin Li, Qiang Li, Chaoqin Ren, Wenxia Sun, Yanfei Huang

**Affiliations:** ^1^ Qinghai-Tibetan Plateau Ethnic Medicinal Resources Protection and Utilization Key Laboratory of National Ethnic Affairs Commission of the People’s Republic of China, Southwest Minzu University, Chengdu, China; ^2^ Institute of Chinese Materia Medica Pharmacology and Toxicology, Sichuan Academy of Chinese Medicine Sciences, Chengdu, China; ^3^ Shimadzu China Co. LTD., Chengdu, China; ^4^ College of Resources and Environment, Aba Teachers College, Wenchuan, China; ^5^ Engineering Research Center for Pharmaceuticals and Equipments of Sichuan Province, School of pharmacy, Sichuan Industrial Institute of Antibiotics, Chengdu University, Chengdu, China

**Keywords:** traditional Chinese medicine, *Meconopsis quintuplinervia* Regel, quality markers, metabolism, LC-MS, content determination

## Abstract

**Background:**

*Meconopsis quintuplinervia* Regel (MQ) is a traditional Chinese medicine (TCM) used for clearing liver and lung heat in Tibetan medicine for over a thousand years. However, the lack of quality markers that correlate with pharmacological actions and absorption *in vivo* limits the safety and efficacy in its clinical application and on the drug market. Furthermore, a universal and rapid method for simultaneously determining quality markers in the MQ crude drug is still lacking.

**Purpose:**

An absorption-based metabolite approach was used to discover and identify the quality markers of MQ. An efficient method based on polarity-switching ultra-performance liquid chromatography triple quadrupole mass spectrometry (UPLC-QQQ-MS/MS) was then established to determine the quality markers.

**Methods:**

The absorbed compounds and metabolites were first characterized using UPLC plus Q-Exactive Orbitrap tandem mass spectrometry (UPLC-Q-Exactive Orbitrap-MS) after giving oral MQ extract to rats. Subsequently, the absorbed compounds and precursors of metabolites correlating with the hepatocyte protection activity were screened as quality markers. Finally, a polarity-switching UPLC-QQQ-MS/MS method was developed for the quantitative analysis of the MQ crude drug, enabling the detection of quality markers in both negative and positive ion modes in a single run. The MS^2^ characteristics of target compounds were investigated to select appropriate product ions.

**Results:**

A total of 13 absorbed compounds and 30 metabolites were characterized. Among these, nine compounds, including five absorbed compounds and four precursors of metabolites intimately correlated with hepatocyte protection activity and absorption *in vivo*, were considered the quality markers of MQ in the current study. The quantification of quality markers was conducted using an Acquity UPLC HSS T3 (2.1 × 100 mm, 1.8 μm) column, the mobile phase consisting of acetonitrile and 0.1% formic acid solution (containing 10 mmol/L ammonium acetate). The validated UPLC-QQQ-MS/MS method was successfully applied to quantify the quality markers in the MQ crude drug.

**Conclusion:**

We defined the quality markers and established a universal and rapid method for simultaneously determining the quality markers of MQ, which will be helpful for further investigation of the quality evaluation of MQ in clinical application and the drug market.

## 1 Introduction

*Meconopsis quintuplinervia* Regel (MQ) is a valuable botanical drug belonging to the Papaveraceae family, distributed only in the Qinghai–Tibetan plateau (Luo et al., 1984). It has been used as a traditional Chinese medicine (TCM) named *Herba Meconopsis* with a long clinical history dating back to the eighth century in the Tibetan book *Yue Wang Yao Zhen* and recorded in the Tibetan masterpieces *The Four Medical Tantras* and the *Jing Zhu Materia Medica*. It has traditionally been used for clearing liver and lung heat (Guo et al., 2016). Given its significant and reliable biological activities, MQ is widely used in more than 100 prescriptions of Tibetan medicine and 31 marketed prescription drugs, such as *Ershiwuwei Luronghao Wan*, *Shiqiwei Hanshuishi Wan*, and *Shibawei Niuhuang San* (Luo et al., 1984; Wang and Ma, 2009; Guo et al., 2016). Previous studies have shown that the pharmacological effect of MQ may be attributed to its chemical compounds such as flavonoids (Shang et al., 2002; Shang et al., 2006b), alkaloids (Shang et al., 2003; Wu et al., 2007), and phenolics (Shang et al., 2006a). The total flavonoid and phenolic fraction extracted from MQ exhibit an obvious hepatoprotective effect on experimental liver damage and hepatic fibrosis, which significantly decreases alanine aminotransferase (ALT), aspartate aminotransferase (AST), and malondialdehyde (MDA) levels, while increasing superoxide dismutase (SOD), catalase (CAT), and reduced glutathione (GSH) levels in carbon tetrachloride (CCl_4_)-induced liver damage mice. Additionally, these fractions reduce the levels of hydroxyproline and collagen in liver tissue, as well as liver fibrosis indicators such as hyaluronic acid, type III procollagen, type IV collagen, and laminin in rats with liver fibrosis (He et al., 2012; Wang et al., 2013a; Wang et al., 2013b). Total alkaloids extracted from MQ exhibit a strong anti-inflammatory effect on LPS-induced acute inflammation in mouse models. This is related to MQ’s ability to inhibit the expression of the pro-inflammatory cytokines tumor necrosis factor-α (TNF-α) and Interleukin-6 (IL-6) and the production of nitric oxide (NO) and to reduce inducible nitric oxide synthase (iNOS) activity (Yu et al., 2021). Kong et al. (2022) employed ultra-performance liquid chromatography plus Q-Exactive Orbitrap tandem mass spectrometry (UPLC-Q-Exactive Orbitrap/MS) and network analysis to analyze the active compounds acting against liver fibrosis. They found that MQ presumably exerted an anti-liver fibrosis effect through luteolin, isorhamnetin, quercetin, apigenin, kaempferide, amurine, 2-methylflavinantine, and allocryptopine on RAC-alpha serine/threonine-protein kinase (AKT1), proto-oncogene tyrosine-protein kinase Src (SRC), transcription factor Jun (JUN), epidermal growth factor receptor (EGFR), and other core targets, and regulated the phosphatidylinositol 3-kinases/protein kinase B (PI3K/AKT), forkhead box protein O (FoxO) and other signaling pathways (Kong et al., 2022).

Traditionally, MQ has primarily been obtained from wild sources, and several species of *Meconopsis* with blue flower were used as *Herba Meconopsis* in clinics and the medical industry. It is difficult to guarantee the clinical efficacy and safety of MQ. Furthermore, the “Pharmaceutical Standards of the Ministry of Health of the People’s Republic of China” for *Herba Meconopsis* uses only morphological and microscopic identification for the quality control of MQ (Chinese Pharmacopoeia Commission, 1995). However, these quality control methods do not reflect MQ’s clinical efficacy or pharmacological activity (Li Y. Z. et al., 2019; Zhang et al., 2019). With the discovery of new compounds and deeper research into the pharmacological activity of MQ, its quality control in clinical application and the market has become more important. It is thus necessary to discover scientific and reasonable quality markers for the quality control of MQ. “Quality markers” are several specific chemical compounds of botanical drugs and their products that are closely related to therapeutic effects and absorption *in vivo* and could be used for qualitative and quantitative analysis (Liu et al., 2016; Liu et al., 2018; Li Y. Z. et al., 2019). In recent years, some studies of quality markers have focused on characterizing the chemical compound–bioactivity association (Wei et al., 2021; Li Y. L. et al., 2022; Li et al., 2024) and prototype-based pharmacokinetic *in vivo* absorption (Li et al., 2018; Duan et al., 2020). These have been the general approach for the analysis of quality markers. However, some studies have shown that TCM metabolism is a crucial link between pharmacological activity *in vivo* and phytochemistry *in vitro*. In brief, the absorbed compounds and metabolites of TCM *in vivo* are the ultimate material basis for the expression of the efficacy of TCM (Xu et al., 2022). Some of the precursors of the metabolites, which are the effective forms of TCM, can be used as quality markers for its quality control (Zhang et al., 2022). This strategy based on metabolites, including absorption compounds and metabolites *in vivo*, we term “absorption-based metabolite strategy”. Unfortunately, few studies have focused on absorption-based metabolite strategy for quality evaluation research into TCM; it may be a powerful approach, and this is the first study of the quality markers of MQ.

Moreover, many methods have been developed to quantify one or several compounds as quality indicators in the MQ raw material. For example, Yue et al. (2010) determined four major flavonoids in hydrolyzed MQ samples by high-performance liquid chromatography (HPLC) coupled with diode array detection. Shi et al. (2011) established an HPLC method for determining one alkaloid in MQ raw material by HPLC. However, for the complex chemical compounds in MQ, the established quantification methods developed by HPLC were limited in their application for the quality control of multiple compounds in varying polarity with long running times and low sensitivity (Jiao et al., 2019). It is thus necessary to establish a universal and rapid method for the simultaneous determination of quality markers in MQ crude drug.

Ultra-performance liquid chromatography triple quadrupole mass spectrometry (UPLC-QQQ-MS/MS) is a widely utilized technology with high separation capability and sensitivity for quantification quality markers in TCM (Li X. Y. et al., 2022). The multiple reaction monitoring (MRM) mode of UPLC-QQQ-MS/MS is a powerful technology for screening specified molecular ion-to-product transitions to quantify quality markers in a specified condition which can effectively avoid interference peak (Jiao et al., 2019; Sun et al., 2023). More importantly, the polarity-switching function in UPLC-QQQ-MS/MS can simultaneously detect different polarity compounds in negative and positive ion modes in a single run (Shen et al., 2022). Therefore, UPLC-QQQ-MS/MS enables the simultaneous determination of different polarity quality markers of MQ simultaneously, sensitively, and rapidly.

In the current study, we explored MQ to discover, identify, and quantify the quality markers by an absorption-based metabolite strategy. First, we explored the metabolism of MQ extract in rats using UPLC-Q-Exactive Orbitrap/MS technology and elucidated the absorbed compounds and metabolites and the metabolic pathways of MQ compounds. Second, we screened the absorbed compounds and the precursors of metabolites that correlated with hepatocyte protection activity, and we validated by reported experimentation these absorbed compounds and the precursors of metabolites *in vivo* that were used as quality markers. Finally, an efficient method based on polarity-switching UPLC-QQQ-MS/MS technology for determining quality markers in MQ was established to determine the quality marker in MQ crude drug. To our knowledge, this is the first study to discover the quality markers of MQ based on the metabolism of absorbed compounds *in vivo*. Our research will be helpful for screening the quality markers of MQ and providing a useful perspective for discovering the quality markers of other TCMs.

## 2 Materials and methods

### 2.1 Chemical reagents

**FIGURE 1:**
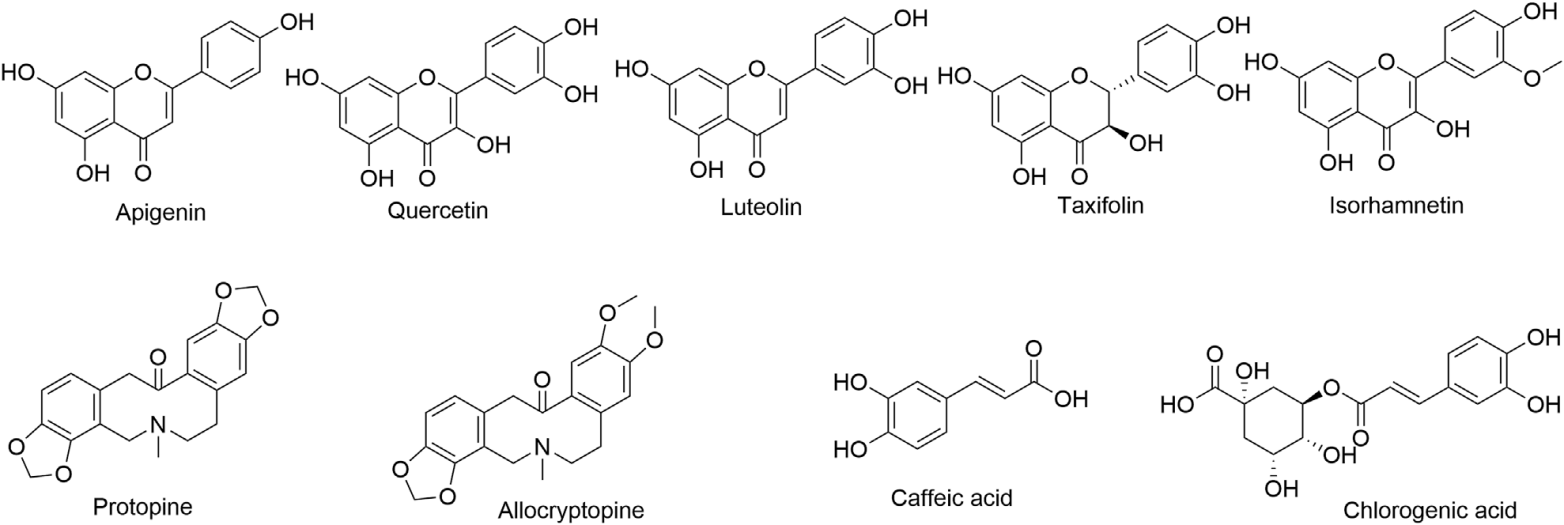
Chemical structures of the nine investigated compounds in MQ.

Quercetin (Lot: MUST-22042012), luteolin (Lot: MUST-22072315), apigenin (Lot: MUST-22111214), taxifolin (Lot: MUST-22101411), isorhamnetin (Lot: MUST-22082703), caffeic acid (Lot: MUST-22062118), chlorogenic acid (Lot: MUST-22111711), allocryptopine (Lot: MUST-22031114), and protopine (Lot: MUST-22062118) with purity ≥98% were purchased from Chengdu MUST Bio-technology Co., Ltd (Chengdu, China). Their structures are shown in Figure 1. HPLC-grade acetonitrile and formic acid were sourced both from Thermo Fisher (Waltham, MA, United States). Ultrapure water was purified at 18.2 MΩ using PERSEE GWB-1E ultrapure water system (Beijing, China). Other reagents were of analytical grade and were purchased locally.

### 2.2 Plant materials

Ten batches of MQ crude drug samples were collected or purchased from Qinghai, Gansu, and Sichuan, China. They were identified by Professor Yuan Liu and Dr. Yanfei Huang as *M. quintuplinervia* Regel (Supplementary Table S1). The voucher specimens of these samples were deposited at the Tibetan, Qiang, and Yi medicinal resources herbarium, Qinghai–Tibetan Plateau Ethnic Medicinal Resources Protection and Utilization Key Laboratory of the National Ethnic Affairs Commission of the People’s Republic of China, Southwest Minzu University.

MQ extract was prepared as per our previous study (Kong et al., 2022). MQ powders (1000 g) were mixed with 70% ethyl alcohol (*v*/*v*) (8 L) and extracted by heating reflux extraction for 1.5 h. This step was repeated three times. The extract was then filtered and dried in vacuum at 50°C and treated with D101 macroporous resins. Afterward, 30%, 50%, and 70% ethyl alcohol eluent was collected and mixed, and the extract was concentrated and freeze-dried into a powder.

### 2.3 Animals and metabolic study

Twelve male Sprague–Dawley rats (weighing 180–220 g) were obtained from the Experimental Animal Center of the Sichuan Academy of Chinese Medicine Sciences (animal production license number: SYXK (2018)-100, Chengdu, China). The rats were kept in an environmentally controlled animal room for 7 days, with water and food *ad libitum* twice daily. The rats were randomly divided into two groups—MQ and blank, with six rats per group—and were housed in metabolic cages for 3 days to acclimatize to the facilities prior to the experiments. MQ extract was suspended in 0.5% CMC-Na solution and orally administered to the MQ group at a daily dose of 50 mg/kg. The MQ extract administration lasted for 5 days, once a day at 9:00 a.m. The blank group was orally administered 0.5% CMC-Na solution at the same volume time. These animal experiments were conducted under the guidance of the *Care and Use of Laboratory Animals* published by the US National Institute of Health and were approved by the Biomedical Ethical Committee of Southwest Minzu University (approval no. SWU-202401160).

### 2.4 Biological sample collection and preparation

#### 2.4.1 Urine and feces samples

Urine samples were collected after administration of MQ extract in the drug and blank groups at 0–24 h. The collection tubes for urine samples were pre-filled with a small volume of absolute ethanol as a preservative. After collection in 1 day, the samples were merged and evaporated to dryness under reduced pressure at 50°C using an electro thermostatic blast oven (DS-30H, Shanghai Dengsheng Instrument Manufacturing Co., Ltd., Shanghai, China). The dried samples of the different groups were then extracted with four-fold methanol (*v*/*w*) in an ultrasonic cleaner for 30 min to precipitate the endogenous substances. These samples were centrifuged at 5,000 rpm, and the supernatant was dried in vacuum at 40°C. Subsequently, each 1.0 g residue of the MQ and blank groups was reconstituted in 5.0 mL methanol and filtered through a 0.22-μm nylon filter before UPLC-Q-Exactive Orbitrap/MS analysis.

Feces samples from the drug group and the blank group were collected twice daily from 8:00 am and 8:00 pm and dried immediately at 50°C using an electro-thermostatic blast oven. Thence, pulverized feces samples (1.0 g) of each group were mixed with 5.0 mL methanol and extracted using an ultrasonic bath for 30 min three times. The extracted samples were centrifuged at 5,000 rpm for 15 min, and the supernatant of the two groups was collected and dried in a rotary evaporator at 40°C. Subsequently, 100 mg of the resulting residue for each group was dissolved in 3 mL methanol and filtered through a 0.22-μm nylon filter before UPLC-Q-Exactive Orbitrap/MS analysis.

#### 2.4.2 Plasma samples

On day 5, after 0.5, 1.0, and 1.5 h of the last administration of MQ extract, blood samples of the two groups were collected into heparinized tubes through heart puncture while the rats were anesthetized by pentobarbital sodium (i.p. 70 mg/kg body weight). Two rats were sacrificed at one time point, and blood samples collected at the same time point from each of the two groups were combined into one sample. These blood samples were centrifuged at 5,000 rpm and 4°C for 10 min to obtain plasma. Next, 6 mL of the two groups of plasma samples (mixed from the three time points, 2 mL per time point) was supplemented with 24 mL methanol and ultrasonically extracted for 30 min to precipitate the protein. The extraction mixture of the drug and blank groups was then centrifuged at 5,000 rpm for 15 min. After that, the supernatant was condensed in a vacuum at 40°C, and the residue was dissolved in 300 μL methanol. Finally, the prepared samples were centrifuged at 15,000 rpm using a Sorvall ST 16R centrifuge (Thermo Fisher Scientific Inc., United States) for 10 min before UPLC-Q-Exactive Orbitrap/MS analysis.

#### 2.4.3 Organ samples

After collecting blood samples from the two groups, the main organs, including the brain, heart, lung, liver, kidney, intestine, and spleen, were rapidly removed from the rats, flushed with pre-cooled normal saline, and frozen at −80°C before further processing. Each of the organs was homogenized in fourfold (volume/wet weight) pre-cooled normal saline. Eightfold methanol was then added to 6 mL homogenate, and extraction was performed in an ultrasonic bath for 30 min. The extraction mixtures of the two groups were centrifuged at 5,000 rpm for 15 min. Next, the supernatant was condensed in a vacuum at 40°C, and the residue was dissolved in 1 mL methanol and filtered through a 0.22-μm nylon filter before UPLC-Q-Exactive Orbitrap/MS analysis.

### 2.5 MQ sample preparation and standard solution

The primary stock solutions of 1,000 μg/mL quercetin, luteolin, apigenin, taxifolin, isorhamnetin, caffeic acid, chlorogenic acid, allocryptopine, and protopine were dissolved in 70% methanol. A series of working standard solutions were prepared by diluting the primary stock solution with 70% methanol. All solutions were stored at 4°C before analysis.

The dried MQ crude drugs were finely powdered in a grinder. One gram of the powder was accurately weighed and then extracted in 70% methanol (75 mL) with the help of heating reflux in 30 min. Afterward, the supernatants were filtered through a 0.22-μm membrane filter before analysis.

### 2.6 UPLC-Q-Exactive Orbitrap/MS analysis conditions

The UPLC-Q-Exactive Orbitrap/MS analyses were performed on a Thermo Scientific Vanquish Flex UPLC coupled with a Q-Exactive Orbitrap Mass analyzer (Thermo Fisher Scientific Inc., United States). The LC-HRMS data were processed by Xcalibur. Chromatography separation was performed on an ACQUITY HSS T3 column (100 × 2.1 mm, 1.8 μm) (Waters, United States) protected with an ACQUITY HSS T3 VanGuard pre-column (2.1 × 5 mm, 1.8 μm) (Waters, United States) at 30°C. The mobile phase consisted of 0.1% formic acid solution (A) and acetonitrile (B), and the gradient elution program was as follows: 5%–10% B at 0–10 min; 10%–20% B at 10–35 min; 20%–27% B at 35–40 min; 27%–33% B at 40–45 min; 33%–55% B at 45–50 min; 55%–90% B at 50–55 min. The flow rate was 0.2 mL/min, and the injection volume was 2 μL.

High-resolution mass spectra analysis conditions were as follows: the data were collected in electrospray ionization (ESI) mode with mass range of *m*/*z* 100–1000 Da of MS^1^ and *m*/*z* 50–1000 Da of MS^2^ in both positive and negative detection modes, with a mass resolution of 75,000. The capillary temperature was 320°C, interface voltage was 3.0 kV (−) and 3.5 kV (+), auxiliary gas heater temperature was 350°C, auxiliary gas flow rate was 3 L/min, and sheath gas flow rate was 12 L/min.

### 2.7 Metabolite characterization

Data analysis was conducted using Xcalibur software. The MQ metabolite extract in the rats was screened as per Li et al. (2017). The structural elucidation strategy of metabolites was conducted in accordance with Li et al. (2020), Zhang et al. (2022), and Su et al. (2023). First, the absorbed compounds were identified by comparison with the previously reported UPLC-Q-Exactive Orbitrap/MS analysis results of MQ (Kong et al., 2022). Second, the skeleton structure of the metabolites was identified by comparing the MS^1^ and MS^2^ data with references or elucidating the chemical structures from online databases, such as SciFinder, PubChem, and ChemSpider. Finally, the metabolic reaction type was confirmed by characteristic mass differences, such as 14.01 Da (CH_2_), 2.01 Da (H_2_), 176.03 Da (C_6_H_8_O_6_), and 79.95 Da (SO_3_), respectively denoting methylation, hydrogenation, glucuronidation, and sulfation metabolic reactions.

The absorbed compounds and metabolites were characterized through the analytical methods described above. Subsequently, the precursors of the MQ metabolites were obtained from the proposed metabolic pathways. Then, the quality markers were screened from the absorbed compounds and the precursors of metabolites were correlated with the hepatocyte protection activity, which was validated by reported experimental validation.

### 2.8 UPLC-QQQ-MS/MS analysis conditions

The UPLC-QQQ-MS/MS assay was performed on an LCMS 8050 triple quadrupole mass spectrometer coupled to the Nexera UPLC with data analysis software Labsolutions LC-MS (Shimadzu, Japan). The column used Acquity UPLC HSS T3 (2.1 × 100 mm, 1.8 μm, Waters) at a column temperature of 40°C. Mobile phase A was acetonitrile, and mobile phase B was 0.1% formic acid solution (containing 10 mmol/L ammonium acetate). The gradient elution procedure was: 5%–60% A at 0–6.5 min; 60%–95%A at 6.5–6.6 min; 95% A at 6.6–8 min; 95%–5%A at 8–8.01 min; 5% B at 8.01–10 min. The flow rate was 0.35 mL/min. Mass spectrometry data were obtained by MRM mode in both positive and negative modes with ESI source. The flow rate of the nebulizer was 3 L/min, drying gas flow rate was 10 L/min, interface temperature was set at 300°C, DL temperature was 250°C, and the heating block temperature was 300°C. The MRM analysis conditions of each target compound for simultaneous analysis are summarized in Table 1.

**TABLE 1:** UPLC-QQQ-MS/MS MRM conditions for nine quality markers.

Name	Ion mode	Retention time (min)	Precursor ion (Q1, *m*/*z*)	Product ion (Q3, *m*/*z*)	Collision energy (eV)	Q1 pre bias	Q3 pre bias
Protopine	+	4.20	354.1	188.1	−31	−13	−12
Allocryptopine	+	4.45	370.1	188.1	−31	−19	−21
Caffeic acid	-	3.37	179.0	135.1	25	20	24
Taxifolin	-	4.16	303.0	285.1	21	21	29
Luteolin	-	5.02	285.0	133.1	25	30	24
Quercetin	-	5.07	301.0	151.1	18	14	29
Apigenin	-	5.55	268.9	117.1	24	18	23
Chlorogenic acid	-	2.90	353.0	191.1	41	13	18
Isorhamnetin	-	5.73	315.0	299.9	28	22	18

### 2.9 Method validation of the established UPLC-QQQ-MS/MS assay

In accordance with the guidelines for the analytical method verification of the Chinese Pharmacopoeia, 2020, Volume 4 (Pharmacopoeia Commission of PRC, 2020), various factors such as linear regression, precision, recovery, limit of detection (LOD), and limit of quantification (LOQ) were investigated. The calibration curve for each target compound was constructed by more than five different concentrations of mixed standard solutions. The sensitivity of the proposed method was evaluated by establishing LOD and LOQ, which were given by concentrations with signal-to-noise ratios (S/N) of 3:1 and 10:1, respectively. Recovery experiments were conducted through six samples of the same MQ sample and spiked authentic standards in MQ sample directly.

## 3 Results and discussion

### 3.1 Metabolite characterization of MQ extract *in vivo*

**FIGURE 2:**
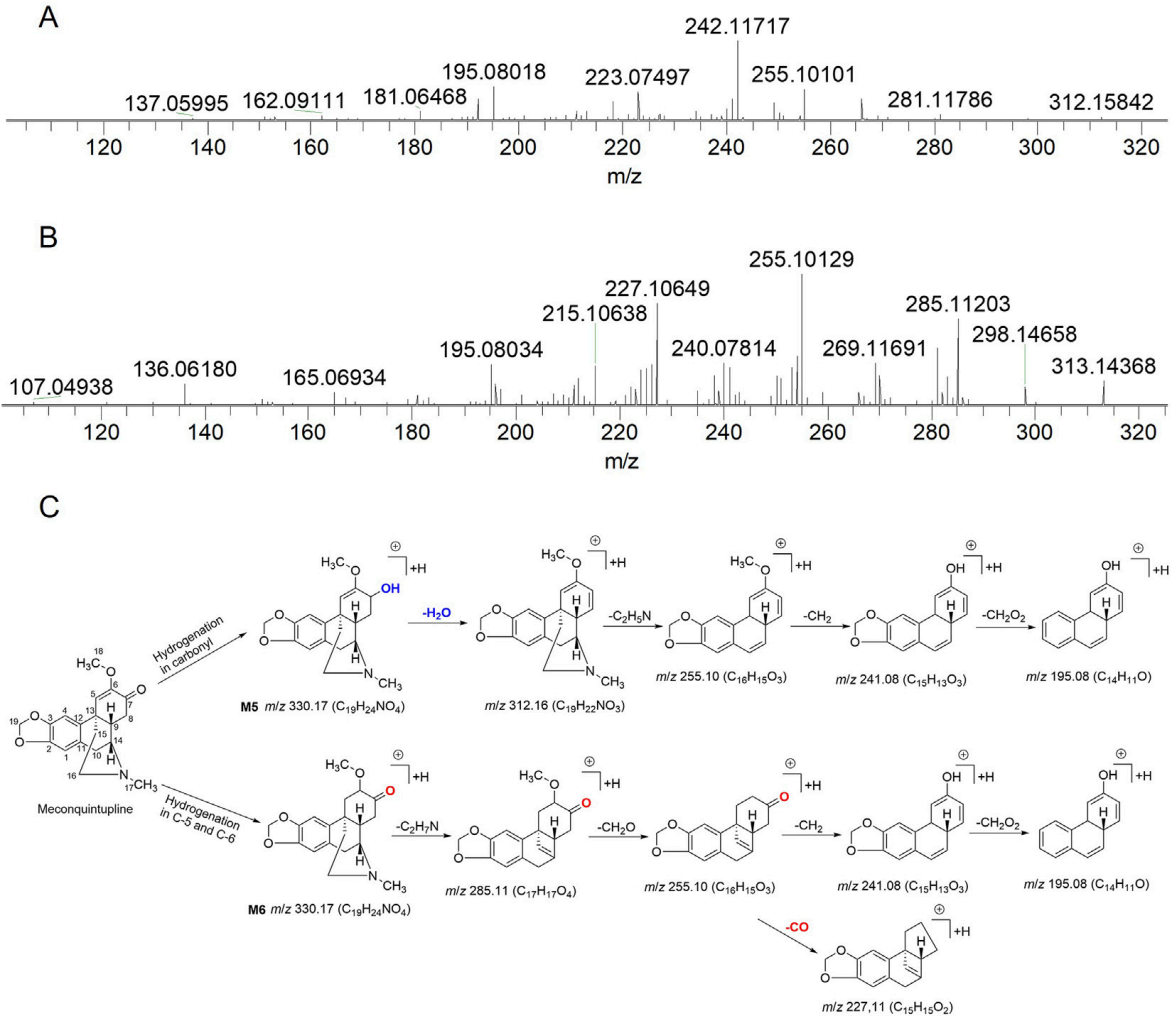
MS^2^ spectra of **M5 (A)** and **M6 (B)** and proposed fragmentation pathway of **M5** and **M6 (C)**.

A total of 13 absorbed compounds (including seven alkaloids, three phenolic acids, two flavonoids, and one other constituent) and 30 metabolites were found in rat urine, feces, plasma, brain, heart, lung, liver, kidney, intestine, and spleen samples. The metabolites consisted of seven phase-I and 23 phase-II metabolites; the chemical structure of these metabolites included 13 alkaloids, 10 flavonoids, and 7 phenolic acids. According to the metabolic reactions, the 23 phase-II metabolites were divided into four groups: methylated metabolites (8), acetylated metabolites (2), sulfated metabolites (9), and glucuronidated metabolites (4). Detailed information of the identification of absorbed compounds, metabolites, and metabolic reactions and the MS data are summarized in Tables 2 and 3; the base peak chromatograms and the distribution of metabolites in main rat organs are shown in Supplementary Figures S1–S10 and Supplementary Tables S2, S3. The structural elucidation of a group of metabolites relevant to meconquintupline is presented below. **M5** and **M6** exhibited [M + H]^+^ at *m*/*z* 330.17, their molecular formula was predicted to be C_19_H_23_NO_4_, and the ring double bond (RDB) was 8. Compared with meconquintupline (MW: 318 Da, MF: C_19_H_21_NO_4_, RDB: 9), the MW of **M5** and **M6** were increased by 2 Da, and the RDB decreased by 1. Therefore, **M5** and **M6** were identified as dihydromeconoquintupline. **M5** and **M6** displayed the same fragment ions of *m*/*z* 271.10, *m*/*z* 255.10, *m*/*z* 241.08, *m*/*z* 195.08, and *m*/*z* 192.10 in MS^2^ spectra. Among these, *m*/*z* 192.10 (C_11_H_14_NO_2_) is a characteristic fragment ion produced by the B ring opening reaction for meconoquintupline (Kong et al., 2022). The loss of the whole bridge ring of **M5** and **M6** produces *m*/*z* 195.08. Furthermore, the dehydration of **M5** was identified, which demonstrated that hydrogenation could occur in the carbonyl at the C-7 position, where the carbonyl was reduced to oxhydryl. In addition, hydrogenation in **M6** may occur in the double bond of C-5 and C-6, given that the loss of carbonyl (C=O) occurred in **M6** but not in **M5**. In particular, the retention time of **M6** (*t*_R_: 18.2 min) was longer than that of **M5** (*t*_R_: 14.0 min), which was consistent with a larger Clog P value meaning a longer *t*_R_ in UPLC. In the present study, the Clog P values of **M5** and **M6** were 1.4 and 1.7, respectively, which demonstrated that **M6** is more hydrophobic than **M5**. Therefore, **M5** and **M6** were identified as 7- dihydro meconoquintupline and dihydromeconoquintupline, respectively. The EICs and MS^2^ data and characteristic fragment ions are shown in Figure 2.

**TABLE 2:** Characterization of absorbed compounds in rats after orally-administered MQ extract.

No.	*t*_R_/min	ESI-MS/(*m*/*z*)	Diff (ppm)	Formula	HR-MS/MS characteristic ion	Identification
F1	23.46	163.03891 [M-H]^-^	−0.061	C_9_H_8_O_3_	119.04887, 117.03339, 93.03315	*p*-coumaric acid
F2	7.49	167.03375 [M-H]^-^	−0.809	C_8_H_8_O_4_	123.04378, 121.02814	Homoprotocatechuic acid
F3	14.34	179.03377 [M-H]^-^	−0.644	C_9_H_8_O_4_	135.04387, 117.05267, 109.02795, 93.03318	Caffeic acid
F4	47.56	269.04510 [M-H]^-^	2.416	C_15_H_10_O_5_	N	Apigenin
F5	44.95	315.05084 [M-H]^-^	2.891	C_16_H_12_O_7_	300.02710, 272.03232, 243.02901, 151.00235, 107.01301	Isorhamnetin
F6	49.04	329.06638 [M-H]^-^	−0.573	C_17_H_14_O_7_	243.02890, 227.03416, 215.03323, 203.03403, 199.03900	Preussiafuran B
F7	21.44	326.13858 [M + H]^+^	−0.105	C_19_H_19_NO_4_	295.09607, 283.09607, 280.07281, 265.08530, 237.09061, 205.06464, 191.09442, 162.09116, 145.08817, 121.50990	Reframoline
F8	13.15	328.15424 [M + H]^+^	−0.095	C_19_H_21_NO_4_	N	Meconquintupline
F9	18.71	342.16986 [M + H]^+^	−0.125	C_20_H_23_NO_4_	297.11194, 282.08850, 265.08572, 250.06212, 237.09076, 222.06754, 191.08539, 166.07793	*O*-methylflavinantine
F10	32.04	354.13312 [M + H]^+^	2.629	C_20_H_19_NO_5_	N	Protopine
F11	33.81	370.16498 [M + H]^+^	−0.998	C_21_H_23_NO_5_	N	Allocryptopine
F12	29.77	372.14423 [M + H]^+^	0.178	C_20_H_21_NO_6_	N	Hydroxylated dihydroprotopine
F13	27.12	384.14383 [M + H]^+^	−0.869	C_21_H_21_NO_6_	N	Hydroxylated oxypseudopalmatine

Note: N, The MS^2^ characteristic ion was not detected.

**TABLE 3:** Characterization of identified metabolites in rats after orally administrated MQ extract.

No.	*t*_R_/min	ESI-MS/(*m*/*z*)	Formula	HR-MS/MS characteristic ion	Identification	Reference
M1	48.09	271.06091 [M-H]^-^	C_15_H_12_O_5_	227.07085, 201.05508, 177.01820, 151.00240, 11 9.04883, 107.01244	Hydrogenated apigenin	Li et al. (2019a)
M2	44.92	317.02969 [M-H]^-^	C_15_H_10_O_8_	299.01920, 271.02444, 255.02942, 227.03430, 178.99741, 151.00237	Hydroxylated quercetin	Qin et al. (2017)
M3	31.27	355.10318 [M-H]^-^	C_16_H_20_O_9_	191.05623, 173.08076, 135.04387, 111.00742	Hydrogenated chlorogenic acid	Liu et al. (2022)
M4	31.8	206.08102 [M + H]^+^	C_11_H_11_NO_3_	185.06320, 162.95728, 160.07561, 145.05209, 133.06476, 117.05734, 103.05454	Demethylated oleracein E	Kong et al. (2022)
M5	13.99	330.16974 [M + H]^+^	C_19_H_23_NO_4_	312.16016, 281.11758, 271.09692, 269.11707, 266.09323, 255.10144, 249.09091, 242.11722, 241.08600, 223.07521, 218.07245, 195.08040, 192.10197, 162.09160	7- dihydro meconoquintupline	Kong et al. (2022)
M6	18.24	330.16965 [M + H]^+^	C_19_H_23_NO_4_	285.11172, 282.12543, 271.09741, 269.11673, 255.10107, 240.07860, 227.10632, 215.10646, 195.08035, 192.10233, 181.06470, 165.06911, 136.06157, 107.04935	Dihydromeconoquintupline	Kong et al. (2022)
M7	17.06	298.14334 [M + H]^+^	C_18_H_19_NO_3_	298.14355, 281.11691, 269.11685, 237.09029, 223.07532, 210.10393, 192.10173, 176.07048, 161.08339, 146.05991, 134.09644	Demethoxylated meconquintupline	Kong et al. (2022)
M8	12.98	193.04955 [M-H]^-^	C_10_H_10_O_4_	178.02600, 137.02306, 121.02816	Methylated caffeic acid	Shi et al. (2019)
M9	9.83	258.99130 [M-H]^-^	C_9_H_8_O_7_S	135.04382	Caffeic acid sulfate isomer 1	Shi et al. (2019)
M10	11.52	258.99127 [M-H]^-^	C_9_H_8_O_7_S	179.03387, 135.04382, 107.04877	Caffeic acid sulfate isomer 2	Shi et al. (2019)
M11	12.97	258.99133 [M-H]^-^	C_9_H_8_O_7_S	135.04387, 107.04881	Caffeic acid sulfate isomer 3	Shi et al. (2019)
M12	4.34	277.00186 [M-H]^-^	C_9_H_10_O_8_S	197.04460, 153.05457, 123.00743	Hydrogenated and hydroxylated caffeic acid sulfate	Shi et al. (2019)
M13	12.41	355.06662 [M-H]^-^	C_15_H_16_O_10_	179.03387, 135.04384, 113.02302	Caffeic acid glucuronide	Shi et al. (2019)
M14	39.94	357.05829 [M-H]^-^	C_18_H_14_O_8_	163.07518, 137.05927, 135.08078	Acetylated isorhamnetin	Ni et al. (2023)
M15	45.69	379.01248 [M-H]^-^	C_16_H_12_O_9_S	299.05566, 284.03220, 256.03726, 211.03970, 151.00250	Methylated kaempferide sulfate	Yang et al. (2023)
M16	38.53	475.08737 [M-H]^-^	C_22_H_20_O_12_	379.06506, 299.05563, 284.03217, 175.02367, 151.00195, 129.01758	Methylated luteolin glucuronide isomer 1	Yang et al. (2023)
M17	39.62	475.08737 [M-H]^-^	C_22_H_20_O_12_	379.06821, 299.05566, 284.03223, 256.03720, 151.00269	Methylated luteolin glucuronide isomer 2	Yang et al. (2023)
M18	20.54	493.09821 [M-H]^-^	C_22_H_22_O_13_	331.08292, 317.06628, 289.07147, 273.07654, 151.00243, 149.02274, 137.02321	Methylated taxifolin glucuronide	Yang et al. (2016a)
M19	34.13	505.09833 [M-H]^-^	C_23_H_22_O_13_	329.06631, 271.02472, 255.02992, 243.02899	Acetylated isoquercetin	Lu et al. (2013)
M20	23.00	234.11227 [M + H]^+^	C_13_H_15_NO_3_	219.08884, 206.11729, 190.08630, 175.07532, 163.06258, 151.07523, 136.05183, 119.04919, 109.21142	Methylated oleracein E isomer 1	Kong et al. (2022)
M21	13.20	234.11223 [M + H]^+^	C_13_H_15_NO_3_	219.08878, 201.07809, 190.08549, 163.06262, 151.07518, 136.05162, 119.04922	Methylated oleracein E isomer 2	Kong et al. (2022)
M22	23.44	234.11226 [M + H]^+^	C_13_H_15_NO_3_	219.08891, 200.07101, 190.08603, 175.07561, 163.06281, 151.07524, 136.05162, 119.04933	Methylated oleracein E isomer 3	Kong et al. (2022)
M23	29.11	248.12794 [M + H]^+^	C_14_H_17_NO_3_	233.10454, 220.09654, 205.07246, 202.13390, 189.09126, 175.07523, 163.06281, 151.07530, 145.06508, 134.02707, 119.04926, 105.06998	Dimethylated oleracein E	Kong et al. (2022)
M24	11.64	300.05350 [M + H]^+^	C_12_H_13_NO_6_S	220.09656, 202.08560, 190.08585, 161.05930, 137.05956, 119.04919	Oleracein E sulfate	Kong et al. (2022)
M25	10.56	396.12808 [M + H]^+^	C_18_H_21_NO_9_	202.08549, 192.10136, 175.07491, 137.05963	Oleracein E glucuronide	Kong et al. (2022)
M26	20.02	342.16968 [M + H]^+^	C_20_H_23_NO_4_	296.10410, 280.10910, 265.08557, 254.09366, 225.09076, 211.07457, 191.09404, 162.09105, 145.08904	Methylated meconquintupline (or *O*-methylflavinantine isomer)	Kong et al. (2022)
M27	10.69	358.16461 [M + H]^+^	C_20_H_23_NO_5_	281.08057, 265.08542, 251.07011, 197.05948, 192.10170, 177.07828, 162.09116, 147.04391, 119.04928	Methoxylated meconquintupline	Kong et al. (2022)
M28	16.93	372.18045 [M + H]^+^	C_21_H_25_NO_5_	326.13937, 285.07581, 257.08038, 242.05727, 211.07536, 192.10185, 177.07919, 153.05536, 125.06001	Methoxylated *O*-methylflavinantine	Kong et al. (2022)
M29	13.31	532.18115 [M + H]^+^	C_26_H_29_NO_11_	356.14877, 206.08105, 189.06909, 188.07025, 151.07523, 135.04384, 119.04931	Hydrogenated protopine glucuronide isomer 1	Huang et al. (2018)
M30	17.69	532.18109 [M + H]^+^	C_26_H_29_NO_11_	189.07030, 188.07054, 165.05449, 151.07524, 135.04398	Hydrogenated protopine glucuronide isomer 2	Huang et al. (2018)

### 3.2 Potential metabolic pathways and precursors of metabolites

**FIGURE 3:**
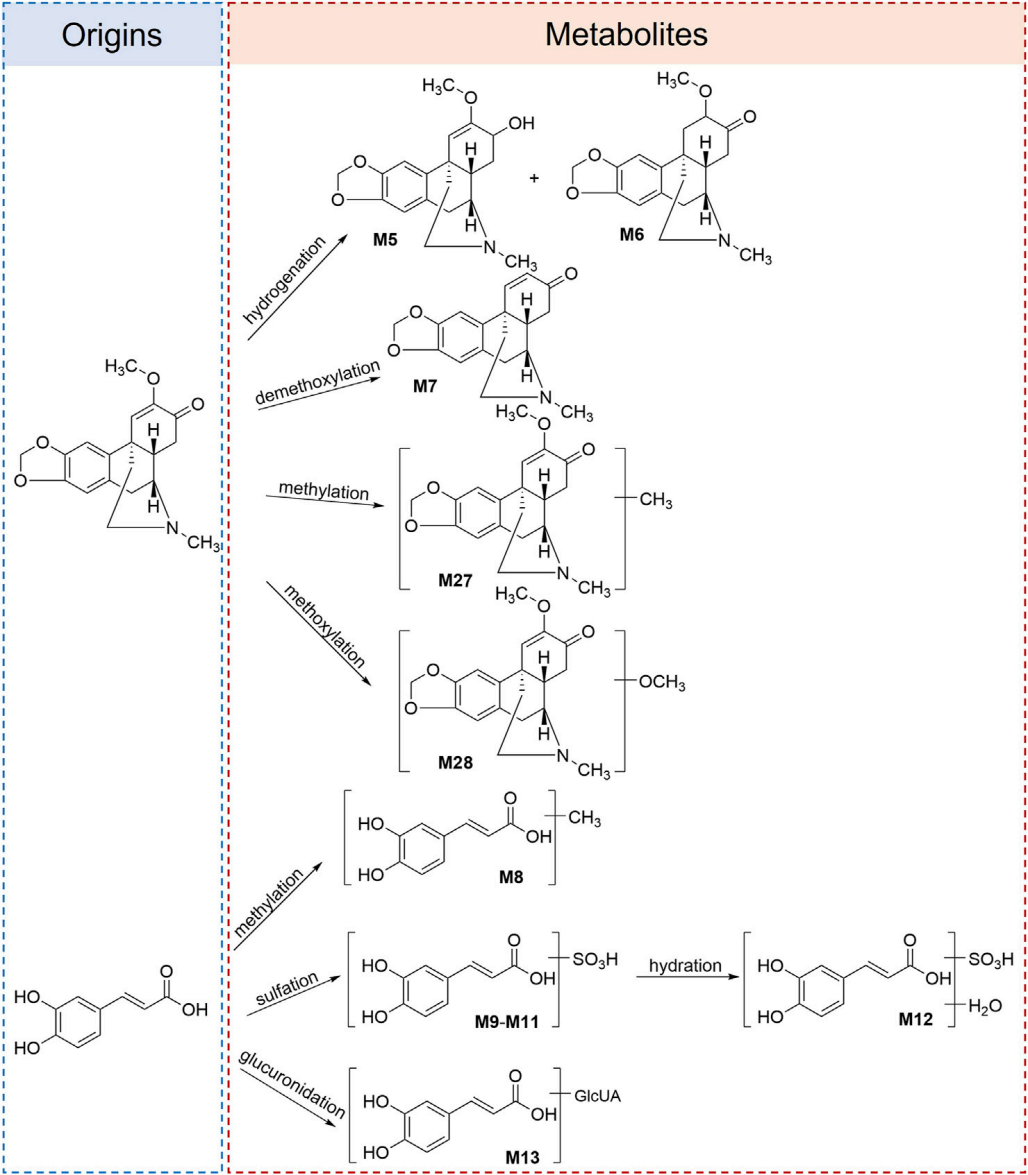
Metabolites and proposed metabolic pathways of meconquintupline and caffeic acid in rats.

In the present study, the proposed precursors of the metabolites were identified by the parent chemical structures and proposed metabolic pathways of the metabolites. As an example, the proposed metabolic pathways of meconquintupline and caffeic acid are presented in Figure 3. The metabolites of 16 MQ compounds were compared with the relevant literature and analyzed to determine their precursors. Finally, we identified seven metabolites derived from oleracein E (**M4**, **M20**, **M21**, **M22**, **M23**, **M24**, and **M25**), six derived from caffeic acid (**M8**, **M9**, **M10**, **M11**, **M12**, and **M13** five from meconquintupline (**M5**, **M6**, **M7, M26**, and **M27**), two from luteolin (**M16** and **M17**), two from protopine (**M29** and **M30**), and eight metabolites derived from eight MQ compounds—apigenin (**M1**), quercetin (**M2**), chlorogenic acid (**M3**), isorhamnetin (**M14**), kaempferide (**M15**), taxifolin (**M18**), isoquercetin (**M19**), and *O*-methylflavinantine (**M28**). Consequently, these absorbed compounds and the proposed metabolite precursors were selected as candidate quality markers.

### 3.3 Hepatocyte protection activity of screened candidate quality markers

In order to verify the reliability of the candidate quality markers screened from absorbed compounds and the precursors of metabolites, the references of experimental validation for hepatocyte protection activity were obtained and analyzed. Apigenin is a common natural flavonoid. Yang et al. (2018) have found that apigenin has the effect of improving hepatocellular carcinoma, one of the mechanisms being apigenin inhibiting cell proliferation and inducing autophagy by inhibiting the PI3K/AKT/mTOR pathway. The data obtained from Rashidi et al. (2023) revealed that isorhamnetin significantly decreases HSC-T6 activation *in vitro* and declines the expression of *COLA1* and *α-SMA* and the protein level of phosphorylated *AKT*, demonstrating that isorhamnetin improves antifibrotic effect by suppressing the PI3K-AKT signaling pathway. Quercetin could ameliorate the lipid metabolism of nonalcoholic fatty liver disease (NAFLD) progression. The mechanism may be that quercetin treatment reduces gene expression, including AKT phosphorylation, inflammation, oxidative stress, and lipid metabolism, and regulates the PI3K/AKT pathway (Sandra et al., 2015). Luteolin also has similar activity. Ahmed et al. (2022) found that luteolin nanoparticles ameliorated NAFLD by regulating the PI3K/AKT/FoxO1 pathway. Taxifolin is an effective flavonoid for liver protection, possibly by inhibiting the activation of hepatic stellate cells and the production of the extracellular matrix (ECM) by regulating the PI3K/AKT/mTOR and TGF⁃β1/Smads pathways to play an anti-hepatic fibrosis role (Liu et al., 2021). Protopine can inhibit the viability of hepatocellular carcinoma cells and trigger apoptosis in a caspase-dependent manner. Additionally, it exerts an anti-hepatocellular carcinoma effect by inducing the accumulation of ROS in hepatocellular carcinoma cells, thereby inhibiting the PI3K/AKT signaling pathway (Nie et al., 2021). Studies have shown that caffeic acid can revert the imbalance of gut microbiota and ameliorate the inflammatory lipopolysaccharide-mediated responses in a NAFLD mouse model, inhibiting the dysregulation of the gene expression of lipid metabolism and improving NAFLD symptoms (Mu et al., 2021). Chlorogenic acid exhibited an effect of improving liver fibrosis in an NAFLD mice model, possibly by inhibiting HSC activation, which promotes mitochondrial biogenesis and reduces ECM production initiated by HMGB1 in hepatic vascular endothelial cells (Miao et al., 2022). Allocryptopine exhibits a significant hepatocyte protection effect, improvement of liver function, and anti-hepatic fibrosis effects in rats with CCl_4_-induced liver fibrosis. It can significantly reduce the expression of liver index, spleen index, AST, ALT, and collagen (CoI, CoIII) in liver tissue (Xiao et al., 2011). Therefore, nine candidate quality markers possess hepatoprotective effects and are absorbable, and these can be detected using reference standards. Consequently, we deduced that apigenin, isorhamnetin, quercetin, luteolin, taxifolin, protopine, allocryptopine, caffeic acid, and chlorogenic acid could be the quality markers of MQ.

The quality markers we have identified in MQ are common compounds, and no specific constituents unique to MQ have been used as quality markers. This poses certain challenges in distinguishing MQ from other botanical drugs of the genus *Meconopsis* during the quality control processes. However, our previous study on *Blumea riparia* and *B. megacephala* found that, although the chemical compounds were similar, their proportions could differ between the two botanical drugs (Su et al., 2023). Other studies have also observed this phenomenon (Kong et al., 2017; Yang et al., 2017). Therefore, we speculate that quality control based on multiple quality markers can achieve differentiation among different botanical drugs of the genus *Meconopsis*.

### 3.4 Method development and quantification of nine quality markers by UPLC-QQQ-MS/MS

#### 3.4.1 Optimization of extraction and UPLC-QQQ-MS/MS conditions

To ensure the extraction efficiency of the compounds being investigated, we investigated as extraction methods different proportions of methanol solvent, material–liquid ratios, and extraction times of MQ samples. The results showed that 70% methanol as the extraction solvent, a material–liquid ratio of 1:75, and heating reflux extraction for 30 min were the best extraction conditions (Supplementary Tables S4–S7). For the chromatographic conditions, according to our previous study (Kong et al., 2022), an acetonitrile–0.1% formic acid solution mobile phase system was preferred for the peak separation of flavonoids and phenolic acids. However, several studies have demonstrated that ammonium acetate could improve the ionization and chromatographic peak shape for alkaloids (Wang et al., 2017). Therefore, acetonitrile-0.1% formic acid solution (containing 10 mmol ammonium acetate) was optimized as the mobile phase system.

**FIGURE 4:**
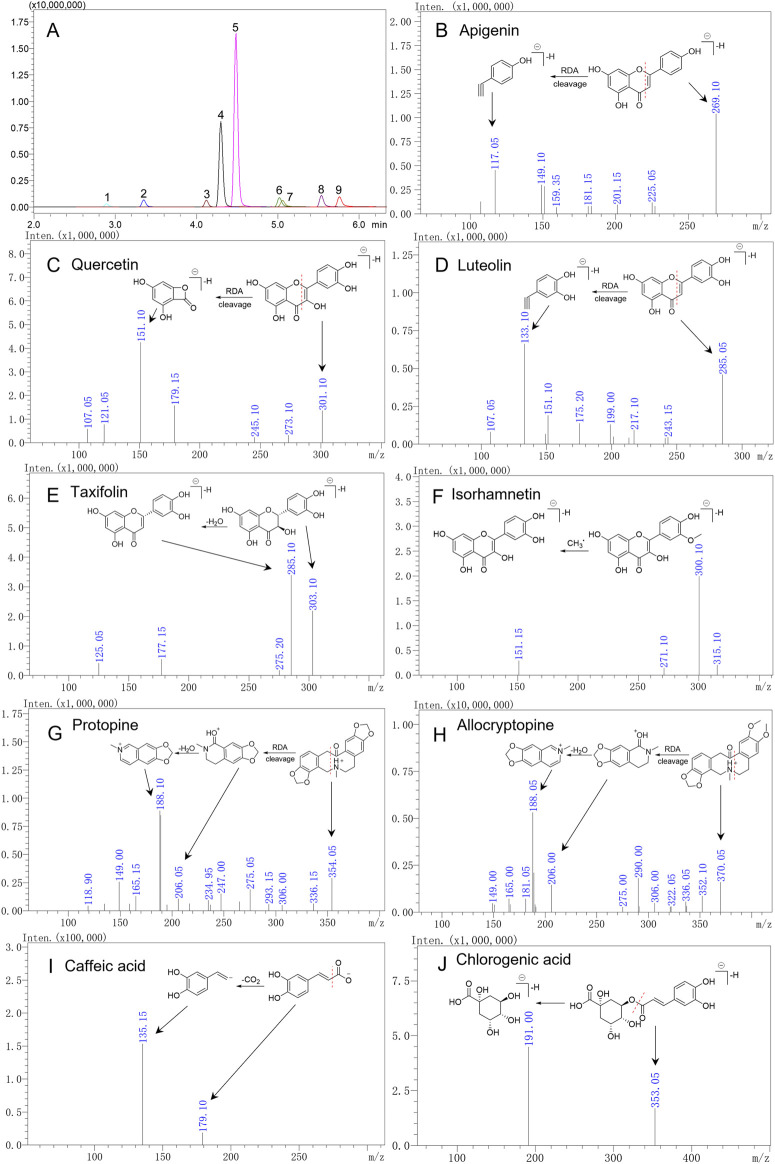
Extracted ion chromatogram of nine quality markers in the MRM mode **(A)** and secondary mass spectra of nine compounds **(B**–**J)**. 1. Chlorogenic acid, 2. caffeic acid, 3. taxifolin, 4. protopine, 5. allocryptopine, 6. luteolin, 7. quercetin, 8. apigenin, and 9. isorhamnetin.

To fully understand the MS characteristics and select appropriate product ions of the nine quality markers, the fragmentation behavior of these compounds was investigated. Among the nine compounds investigated, the chemical structure of apigenin, luteolin, quercetin, and isorhamnetin was that of flavones and flavonols, and they were analyzed in the negative mode. In the MS^2^ spectrogram after CID cracking, the products of Retro–Diels–Alder (RDA) cleavage were characteristic fragments, such as *m*/*z* 117 (^1,3^B^−^) from apigenin, *m*/*z* 133 (^1,3^B^−^) from luteolin, and *m*/*z* 151 (^1,3^A^−^) from quercetin and isorhamnetin. These fragments could be used as product ions in MRM analysis except for isorhamnetin, because the RDA product of isorhamnetin was not the dominant fragment ion. The dominant fragment ion of isorhamnetin was *m*/*z* 300 [M-H-CH_3_^•^]^-•^, which was the product from the loss of CH_3_^•^. Differently from these three compounds where C-2 and C-3 had a double bond, taxifolin is a flavanonol; for the saturated bond in C-2 and C-3, the dehydration product *m*/*z* 285 [M-H-H_2_O]^-^ was the dominant fragment ion, which could be used as product ion in MRM analysis (Figure 4). Protopine and allocryptopine are isoquinoline alkaloids, and they were analyzed in the positive ion mode. The chemical structure of allocryptopine is similar to that of protopine, and the fragmentation pathway is also similar. Protopine and allocryptopine displayed abundant fragment ions of *m*/*z* 336, *m*/*z* 306, *m*/*z* 206, *m*/*z* 188, and *m*/*z* 149 in MS^2^ spectra. In particular, *m*/*z* 206 was a diagnostic fragment ion produced by RDA cleavage from the parent ion; the ion of *m*/*z* 188 was a dehydration product from *m*/*z* 206; *m*/*z* 188 was also the dominant fragment ion in MS^2^ spectra. Therefore, *m*/*z* 188 could be used as product ion in MRM analysis (Figure 4).

Yang W. Y. et al. (2016) and Shen et al. (2022) have sought to simultaneously determine the content of different polarity compounds in TCM. The highly specific MRM technique can accurately quantify the analytes of interest with MS or MS^2^ characteristics in the positive and negative ion modes. Hence, in the present study, polarity-switching used for MRM analysis was selected to analyze MQ samples in order to simultaneously determine flavonoids, phenolic acids, and alkaloids. In MRM analysis, it is crucial to determine the precursor ions of each compound and its product ions. Usually, the precursor ions were determined in Q1 MS mode, and then one to two product ions with higher abundance were selected according to the results of its product ion scanning. Finally, collision voltage (CE) was optimized to ensure the best MRM conditions. Under optimized conditions, nine quality markers were isolated and eluted in 10 min (Figure 4), with detailed parameters listed in Table 1.

#### 3.4.2 Method validation of the developed UPLC-QQQ-MS/MS MRM assay

To evaluate the sensitivity and precision of the established UPLC-QQQ-MS/MS method, the linearity, LOD, LOQ, precision, stability, repeatability, and recovery of the nine target compounds were verified, with the results in Table 4 and Supplementary Tables S8–S10. The standard curves for all nine target compounds showed good linear fit in the range of 100–2000 ng/mL, and the correlation coefficients (*r*) were above 0.99. The LOD and LOQ values of the nine target compounds were 0.81–4.15 ng/mL and 2.47–12.59 ng/mL, respectively, indicating high sensitivity under the optimized chromatographic conditions. Precision was evaluated by the spiked standard at a concentration of 1000 ng/mL. The results showed that the RSD value of each compound varied 0.78–2.97%, indicating good precision. In addition, repeatability was tested for the MQ1 sample in sextuplicate; the concentrations of the nine target compounds were calculated, and the mean repeatability expressed as RSD was 2.49–5.68%. Accuracy was assessed by the MQ1 sample in sextuplicate with a known amount of standard with 100% level that was compared with the concentration of the nine target compounds in the MQ1 sample. Recovery was calculated by the following formula: recovery (%) = (amount detected − original amount)/100%. In the present study, the mean RSD of the recovery test in our developed analytical method was 85.71–103.78%. Finally, stability was examined under the MQ1 sample at 0, 2, 4, 6, 10, and 24 h in triplicate; the results showed that the RSD of the peak area of the nine target compounds was 1.37%–4.79%. These results indicated that the UPLC-QQQ-MS/MS method that we developed was accurate, precise, sensitive, and reliable enough to quantitatively determine simultaneously the nine quality markers in the MQ samples.

**TABLE 4:** Calibration curves, linearity, LOD, and LOQ for nine quality markers.

Name	Retention time (min)	Linear range (ng/mL)	Regression equation	*r*	LOD (ng/mL)	LOQ (ng/mL)	Recovery (%)	RSD (%)
Protopine	4.20	100–2000	*y* = 22,471.0*x*-106242	0.9995	0.81	2.47	97.82	1.03
Allocryptopine	4.45	100–2000	*y* = 20,711.8*x*+10,232.2	0.9994	0.90	2.70	95.17	3.91
Caffeic acid	3.37	100–2000	*y* = 3,346.53*x*+138,348	0.9900	2.86	8.67	85.71	1.46
Taxifolin	4.16	100–2000	*y* = 2,674.22*x*+29,478.6	0.9997	1.80	5.42	90.73	1.50
Luteolin	5.02	100–2000	*y* = 4,120.01*x*+149,437	0.9929	2.41	7.33	90.71	2.49
Quercetin	5.07	100–2000	*y* = 2,997.01*x*+74,321.8	0.9940	3.38	10.23	102.45	3.55
Apigenin	5.55	100–2000	*y* = 4,641.91*x*+78,986.6	0.9965	1.26	3.82	86.65	2.81
Chlorogenic acid	2.90	100–2000	*y* = 1941.8*x*+78,501.0	0.9974	1.97	5.96	93.29	1.74
Isorhamnetin	5.73	100–2000	*y* = 7,363.41*x*-38408.6	0.9957	4.15	12.59	103.77	4.28

#### 3.4.3 Quantification of quality markers in MQ by UPLC-QQQ-MS/MS

The UPLC-QQQ-MS/MS method developed was successfully applied to the quantitative analysis of quality markers belonging to flavonoids, alkaloids, and phenolics in MQ samples collected from different areas at different altitudes of Gansu, Qinghai, and Sichuan provinces in China. All compounds investigated were eluted within 10 min in the polarity-switching positive and negative ion modes of the ESI source. The content of the nine quality markers was detected at the average range of 1.02–137.29 μg/g; among them, luteolin and taxifolin had relatively large amounts in the MQ samples (Table 5). A comparison between the sample collected at the highest altitude (MQ1) and the batches collected at the lowest altitudes (MQ8) revealed a decreasing trend in alkaloid content with increasing altitude; for example, the protopine content in MQ1 was 5.83 μg/g but was 13.14 μg/g in MQ8. In contrast, the flavonoid content exhibited an increasing trend with higher altitudes; for example, the isorhamnetin content in MQ1 was 10.48 μg/g but was 6.22 μg/g in MQ8. Zhang et al. 2010) also found a similar trend in MQ, with the content of quercetin and luteolin significant increasing with rising altitude in the Qinghai Dalijia area. However, in the Lajishan area, there was a trend of first decrease and then increase. Pandey et al. (2018) also reported similar results in alkaloid and flavonoids. They found that the berberine content of *Thalictrum foliolosum* varied inversely with altitude, the flavonoid and phenolic content of *T. foliolosum* increased at higher altitudes, while the content of these compounds may still vary according to growth and season (Pandey et al., 2018).

**TABLE 5:** Content determination results of nine quality markers of MQ samples (μg/g crude drug).

Name	MQ1	MQ2	MQ3	MQ4	MQ5	MQ6	MQ7	MQ8	MQ9	MQ10	Average
Protopine	5.83	5.88	5.69	10.13	6.19	8.96	13.52	13.14	4.88	7.48	8.17 ± 3.00
Allocryptopine	0.55	0.46	2.15	1.92	1.85	1.84	0.48	0.27	0.1	0.58	1.02 ± 0.77
Caffeic acid	21.93	20.66	17.43	16.74	16.42	16.99	19.31	1.45	20.29	1.55	15.28 ± 7.11
Taxifolin	221.86	224.68	84.11	79.47	54.82	80.70	58.33	73.43	276.03	219.45	137.29 ± 82.02
Luteolin	69.83	67.48	19.74	47.28	29.91	48.49	66.97	66.03	105.61	102.93	62.43 ± 26.31
Quercetin	7.97	8.05	5.29	6.82	4.09	6.47	4.27	4.7	16.42	9.72	7.38 ± 3.48
Apigenin	8.70	8.77	2.18	7.03	4.44	6.77	10.33	6.57	14.85	5.26	7.49 ± 3.31
Chlorogenic acid	17.25	16.28	8.87	6.75	27.72	36.27	55.93	4.74	62.30	9.94	24.61 ± 19.61
Isorhamnetin	10.48	10.27	8.31	6.26	8.06	7.76	7.40	6.22	9.23	6.55	8.05 ± 1.47

## 4 Conclusion

In the present study, a total of 13 absorbed compounds (including seven alkaloids, three phenolic acids, two flavonoids, and one other compound) and 30 metabolites of MQ extract were found in rat urine, feces, plasma, brain, heart, lung, liver, kidney, intestine, and spleen samples. The nine absorbed compounds and the precursors of metabolites, luteolin, isorhamnetin, quercetin, apigenin, allocrytopine, taxifolin, protopine, caffeic acid, and chlorogenic acid were related to hepatocyte protection activity, which selected them as the quality markers.

The established UPLC-QQQ-MS/MS quantitative method presented reasonable linearity, precision, accuracy, repeatability, stability and recovery for the nine quality markers. These markers were all detected in MQ crude drug. Among them, luteolin and taxifolin had relatively large amounts in the MQ samples. There are variations in the quality of MQ samples sourced from different areas and altitudes, with the variations presenting a decreasing trend in alkaloids with increasing altitude and an increasing trend in flavonoids with increasing altitude.

This study used an absorption-based metabolite strategy to successfully explore, discover, and identify the quality markers of MQ, and a universal and rapid method based on the polarity-switching UPLC-QQQ-MS/MS technology was established for the simultaneous determination of quality markers in MQ. Our study provides a research strategy to optimize quality markers and more rigorous quality control of TCM.

## Data Availability

The original contributions presented in the study are included in the article/Supplementary Material, further inquiries can be directed to the corresponding author.
