# Supplementary material for: Discovery of quality markers of Meconopsis quintuplinervia based on an absorption-based metabolite approach and rapid quantification using polarity-switching UPLC-QQQ-MS/MS
Source: Front Pharmacol. 2024 Dec 6;15:1474768. doi: 10.3389/fphar.2024.1474768 (PMC11658988; doi:10.3389/fphar.2024.1474768)
Supplement: Supplementary file 1 [file Table1.docx]

Supplementary Material

# Supplementary Tables

Table S1. Sample information of *Meconopsis quintuplinervia*

| No. | Collection location | Altitude (m) | Voucher specimen number |
| --- | --- | --- | --- |
| MQ1 | Baiyu County, Ganzi Tebetan Autonomous Prefecture, Sichuan province, China | 4300 | WM2201 |
| MQ2 | Zhuoda Mountain, Ganzi County, Ganzi Tibetan Autonomous Prefecture, Sichuan province, China | 4422 | WM2202 |
| MQ3 | Dagu Glacier, Heishui County, Aba Tibetan Autonomous Prefecture, Sichuan province, China | 3632 | WM2203 |
| MQ4 | Xiawute Village, Tuyema Township, Henan Mongolian Autonomous County, Huangnan Tibetan Autonomous Prefecture, Qinghai Province, China | 3737 | WM2204 |
| MQ5 | Sailong Village, Zeku County, Huangnan Tibetan Autonomous Prefecture, Qinghai Province, China | 3611 | WM2205 |
| MQ6 | Dariga Mountain, Xunhua Sala Autonomous County, Haidong City, Qinghai Province, China | 3504 | WM2206 |
| MQ7 | Quanzang village, Hualong Hui Autonomous County, Haidong City, Qinghai Province, China | 3284 | WM2207 |
| MQ8 | Daban Mountain, Baha Village, Dongchuan Town, Hui Autonomous County, Menyuan, Qinghai Province, China | 3115 | WM2208 |
| MQ9 | Dalijia mountain, Xunhua county, Haidong City, Qinghai province, China | 3529 | WM2209 |
| MQ10 | Tanglongduo village, Luqu County, Gannan Tibetan Autonomous Prefecture, Gansu Province, China | 3390 | WM2210 |

Table S2. Distribution of absorbed prototype compounds in rats

| No. | Identification | Urine | Feces | Plasma | Intestine | Brain | Lung | Kidney | Heart | Liver | Spleen |
| --- | --- | --- | --- | --- | --- | --- | --- | --- | --- | --- | --- |
| F1 | *p*-coumaric acid | △ | / | △ | △ | / | △ | △ | / | / | △ |
| F2 | Homoprotocatechuic acid | △ | △ | △ | / | / | / | △ | / | / | / |
| F3 | Caffeic acid | / | △ | △ | △ | / | △ | △ | △ | △ | △ |
| F4 | Apigenin | / | / | △ | / | / | / | / | / | / | △ |
| F5 | Isorhamnetin | △ | / | △ | / | / | / | / | / | / | / |
| F6 | Preussiafuran B | △ | / | △ | △ | △ | △ | / | △ | / | △ |
| F7 | Reframoline | △ | △ | △ | △ | △ | △ | △ | △ | △ | △ |
| F8 | Meconquintupline | △ | △ | △ | △ | △ | △ | △ | △ | △ | △ |
| F9 | *O*-methylflavinantine | △ | △ | △ | △ | △ | △ | △ | △ | △ | △ |
| F10 | Protopine | △ | / | / | / | / | △ | △ | △ | △ | △ |
| F11 | Allocryptopine | △ | △ | / | / | / | / | / | / | / | / |
| F12 | Hydroxylated dihydroprotopine | △ | / | / | / | / | / | / | / | / | / |
| F13 | Hydroxylated oxypseudopalmatine | △ | / | / | / | / | / | / | / | / | / |

Note: △: The constituent was detected. /: The constituent was not detected.

**Table S3. Distribution of metabolites in rats**

| No. | Identification | Urine | Feces | Plasma | Intestine | Brain | Lung | Kidney | Heart | Liver | Spleen |
| --- | --- | --- | --- | --- | --- | --- | --- | --- | --- | --- | --- |
| M1 | Hydrogenated apigenin | △ | △ | △ | / | / | △ | △ | △ | △ | / |
| M2 | Hydroxylated quercetin | △ | △ | / | / | / | / | / | / | / | / |
| M3 | Hydrogenated chlorogenic acid | △ | / | / | / | / | / | / | / | / | / |
| M4 | Demethylated oleracein E | △ | △ | △ | △ | / | / | △ | △ | △ | △ |
| M5 | 7- dihydro meconoquintupline | △ | △ | / | / | / | / | / | / | / | / |
| M6 | Dihydromeconoquintupline | △ | △ | / | / | / | / | / | / | / | / |
| M7 | Demethoxylated meconquintupline | △ | / | / | / | / | / | / | / | / | / |
| M8 | Methylated caffeic acid | △ | △ | / | / | / | △ | / | / | △ | / |
| M9 | Caffeic acid sulphate isomer 1 | △ | / | / | / | / | / | / | / | / | / |
| M10 | Caffeic acid sulphate isomer 2 | △ | / | / | / | / | / | / | / | / | / |
| M11 | Caffeic acid sulphate isomer 3 | △ | / | / | / | / | / | / | / | / | / |
| M12 | Hydrogenated and hydroxylated caffeic acid sulphate | △ | / | / | / | / | / | / | / | / | / |
| M13 | Caffeic acid glucuronide | △ | △ | △ | / | △ | △ | △ | △ | / | / |
| M14 | Acetylated isorhamnetin | △ | / | / | / | / | / | / | / | / | / |
| M15 | Methylated kaempferide sulphate | △ | △ | △ | / | / | △ | △ | / | / | △ |
| M16 | Methylated luteolin glucuronide isomer 1 | △ | △ | △ | △ | △ | △ | △ | △ | △ | △ |
| M17 | Methylated luteolin glucuronide isomer 2 | △ | △ | △ | △ | △ | △ | △ | △ | △ | △ |
| M18 | Methylated taxifolin glucuronide | △ | / | / | △ | / | △ | △ | / | △ | △ |
| M19 | Acetylated isoquercetin | △ | / | / | / | / | △ | △ | / | / | / |
| M20 | Methylated oleracein E isomer 1 | △ | △ | / | △ | / | △ | △ | △ | / | △ |
| M21 | Methylated oleracein E isomer 2 | △ | △ | △ | △ | / | / | △ | △ | / | △ |
| M22 | Methylated oleracein E isomer 3 | △ | △ | △ | △ | / | △ | △ | △ | / | △ |
| M23 | Dimethylated oleracein E | △ | △ | / | / | / | △ | △ | △ | / | / |
| M24 | Oleracein E sulphate | △ | / | / | / | / | △ | / | / | / | / |
| M25 | Oleracein E glucuronide | △ | △ | △ | / | △ | △ | △ | △ | / | / |
| M26 | Methylated meconquintupline | △ | △ | / | / | / | / | / | △ | / | / |
| M27 | Methoxylated meconquintupline | △ | △ | / | / | / | / | △ | / | / | / |
| M28 | Methoxylated *O*-methylflavinantine | △ | / | △ | / | / | / | / | / | / | / |
| M29 | Hydrogenated protopine glucuronide isomer 1 | △ | / | △ | / | / | / | / | / | / | / |
| M30 | Hydrogenated protopine glucuronide isomer 2 | △ | / | / | / | / | / | / | / | / | / |

Note: △: The constituent was detected. /: The constituent was not detected.

**Table S4. Results of extraction method (μg/g)**

| Extraction method | Protopine | Allocryptopine | Caffeic acid | Taxifolin | Luteolin | Quercetin | Apigenin | Chlorogenic acid | Isorhamnetin |
| --- | --- | --- | --- | --- | --- | --- | --- | --- | --- |
| Heating reflux extraction | 8.73 | 0.33 | 12.50 | 24.59 | 34.42 | 1.89 | 4.97 | 38.93 | 3.17 |
| Ultrasonic extraction | 7.86 | 0.31 | 6.84 | 12.86 | 28.91 | 1.89 | 5.85 | 39.01 | 5.66 |

**Table S5. Results of different proportions of methanol solvent (μg/g)**

| Proportions of methanol | Protopine | Allocryptopine | Caffeic acid | Taxifolin | Luteolin | Quercetin | Apigenin | Chlorogenic acid | Isorhamnetin |
| --- | --- | --- | --- | --- | --- | --- | --- | --- | --- |
| 30% methanol | 3.03 | 0.16 | 11.63 | 9.48 | / | / | / | 34.51 | 0.14 |
| 50% methanol | 5.27 | 0.22 | 11.05 | 21.32 | 11.15 | 0.01 | 1.63 | 36.66 | 0.86 |
| 70% methanol | 8.73 | 0.33 | 12.50 | 24.59 | 34.42 | 1.89 | 5.14 | 38.93 | 4.19 |
| 100% methanol | 9.21 | 0.23 | 10.87 | 36.97 | 52.42 | 2.24 | 5.57 | 30.26 | 3.98 |

Note: “/” means not detected.

**Table S6. Results of solid-liquid ratio (μg/g)**

| Solid-liquid ratio | Protopine | Allocryptopine | Caffeic acid | Taxifolin | Luteolin | Quercetin | Apigenin | Chlorogenic acid | Isorhamnetin |
| --- | --- | --- | --- | --- | --- | --- | --- | --- | --- |
| 1:25 | 8.53 | 0.37 | 13.63 | 26.23 | 49.38 | 3.4 | 6.65 | 43.94 | 5.73 |
| 1:50 | 12.09 | 0.49 | 17.36 | 49.67 | 62.74 | 1.61 | 9.45 | 55.71 | 9.79 |
| 1:75 | 15.97 | 0.49 | 16.92 | 62.88 | 57.78 | 1.67 | 9.56 | 54.34 | 11.23 |
| 1:100 | 12.68 | 0.44 | 16.39 | 60.73 | 33.01 | 1.58 | 6.53 | 41.39 | 9.02 |

**Table S7. Results of extraction time (μg/g)**

| Extraction time | Protopine | Allocryptopine | Caffeic acid | Taxifolin | Luteolin | Quercetin | Apigenin | Chlorogenic acid | Isorhamnetin |
| --- | --- | --- | --- | --- | --- | --- | --- | --- | --- |
| 0.5 h | 3.87 | 0.15 | 17.88 | 60.04 | 35.20 | 2.26 | 6.45 | 49.36 | 5.92 |
| 1.0 h | 4.49 | 0.14 | 19.50 | 58.26 | 25.81 | 1.74 | 4.53 | 44.16 | 5.34 |
| 1.5 h | 3.81 | 0.15 | 18.42 | 61.85 | 29.93 | 2.07 | 5.19 | 41.67 | 3.53 |

**Table S8. Results of precision**

| No/ Peak area | Protopine | Allocryptopine | Caffeic acid | Taxifolin | Luteolin | Quercetin | Apigenin | Chlorogenic acid | Isorhamnetin |
| --- | --- | --- | --- | --- | --- | --- | --- | --- | --- |
| 1 | 2,178,659 | 2,076,610 | 446,966 | 275,151 | 541,762 | 362,290 | 525,501 | 210,293 | 751,485 |
| 2 | 2,121,295 | 2,059,821 | 450,721 | 275,131 | 512,851 | 350,753 | 530,634 | 202,371 | 755,114 |
| 3 | 2,193,796 | 2,143,704 | 442,837 | 269,293 | 510,222 | 348,123 | 512,038 | 204,223 | 765,445 |
| 4 | 2268676 | 2,100,134 | 450,176 | 272,080 | 517,481 | 334,376 | 524,721 | 198,840 | 763,874 |
| 5 | 2,276,542 | 1,994,538 | 432,811 | 277,365 | 520,014 | 347,271 | 521,093 | 212,375 | 753,734 |
| 6 | 2,122,344 | 2,076,610 | 442,774 | 275,151 | 526,317 | 358,565 | 519,245 | 197,462 | 762,372 |
| RSD (%) | 2.97 | 2.64 | 1.65 | 1.45 | 2.40 | 2.80 | 1.22 | 2.95 | 0.78 |

**Table S9. Results of repeatability**

| No/ Peak area | Protopine | Allocryptopine | Caffeic acid | Taxifolin | Luteolin | Quercetin | Apigenin | Chlorogenic acid | Isorhamnetin |
| --- | --- | --- | --- | --- | --- | --- | --- | --- | --- |
| 1 | 1,547,254 | 147,048 | 1,049,828 | 7,616,831 | 3,678,267 | 367,718 | 609,646 | 528,595 | 1,094,086 |
| 2 | 1,736,281 | 136,496 | 1,183,630 | 7,825,694 | 3,573,966 | 418,179 | 624,772 | 521,732 | 986,852 |
| 3 | 1,687,279 | 138,724 | 1,120,042 | 7,387,272 | 3,749,112 | 414,479 | 644,193 | 557,801 | 1,008,170 |
| 4 | 1,553,552 | 139,322 | 1,100,724 | 8,263,176 | 4,091,444 | 383,726 | 599,300 | 527,364 | 931,089 |
| 5 | 1,558,574 | 142,321 | 1,102,732 | 8,255,578 | 4,103,472 | 396,352 | 619,273 | 519,286 | 987232 |
| 6 | 1,511,832 | 136,194 | 1,049,828 | 7,825,694 | 3,878,342 | 372,231 | 612,297 | 542,174 | 992623 |
| RSD (%) | 5.63 | 2.93 | 4.33 | 4.42 | 5.68 | 5.42 | 2.49 | 2.74 | 5.30 |

**Table S10. Results of** **stability**

| No/ Peak area | Protopine | Allocryptopine | Caffeic acid | Taxifolin | Luteolin | Quercetin | Apigenin | Chlorogenic acid | Isorhamnetin |
| --- | --- | --- | --- | --- | --- | --- | --- | --- | --- |
| 0 h | 1,547,254 | 147,048 | 1,049,828 | 7,616,831 | 3,678,267 | 367,718 | 609,646 | 528,595 | 1,094,086 |
| 2 h | 1,527,254 | 146,273 | 972,323 | 7,639,273 | 3,392,271 | 343,252 | 593,284 | 521,626 | 987,234 |
| 4 h | 1,534,920 | 142,048 | 971,723 | 7,599,377 | 3,312,172 | 334,824 | 582,731 | 516,247 | 988,212 |
| 6 h | 1,529,364 | 146,273 | 962,374 | 7,462,826 | 3,273,864 | 336,672 | 572,837 | 501,274 | 977,238 |
| 10 h | 1,512,744 | 143,834 | 962,848 | 7,379,924 | 3,301,273 | 327,142 | 571,237 | 492,871 | 972,631 |
| 24 h | 1,577,632 | 137,412 | 959,472 | 7,126,217 | 3,212,322 | 329,288 | 560,227 | 487,261 | 969,172 |
| RSD (%) | 1.37 | 2.94 | 3.44 | 3.19 | 4.79 | 4.66 | 3.22 | 4.33 | 4.53 |

# Supplementary Figures


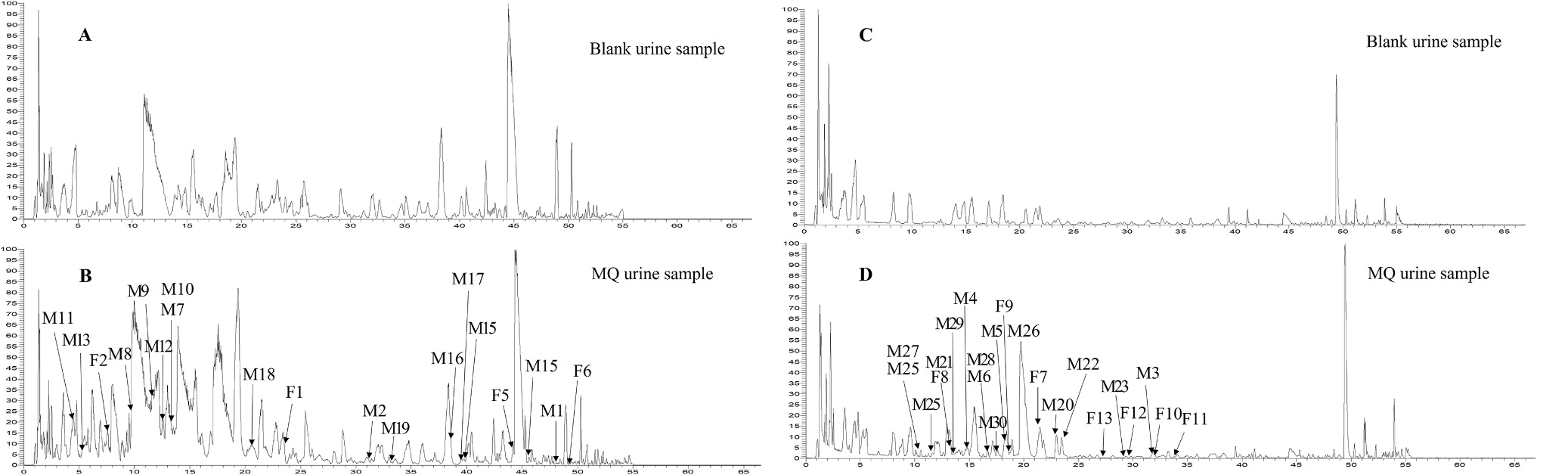


**Supplementary Figure 1.** Base peak chromatograms of absorbed compounds and metabolites in rats’ urine after administration of *Meconopsis quintuplinervia* extract in negative (A and B) and positive (C and D) ion modes.


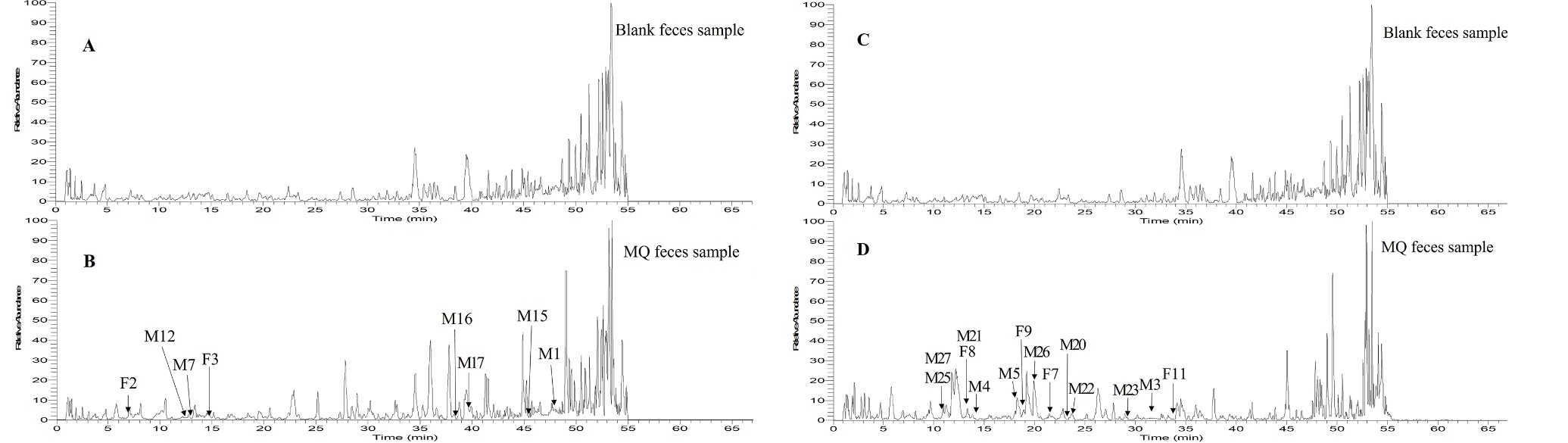


**Supplementary Figure 2.** Base peak chromatograms of absorbed compounds and metabolites in rats’ feces after administration of *Meconopsis quintuplinervia* extract in negative (A and B) and positive (C and D) ion modes.


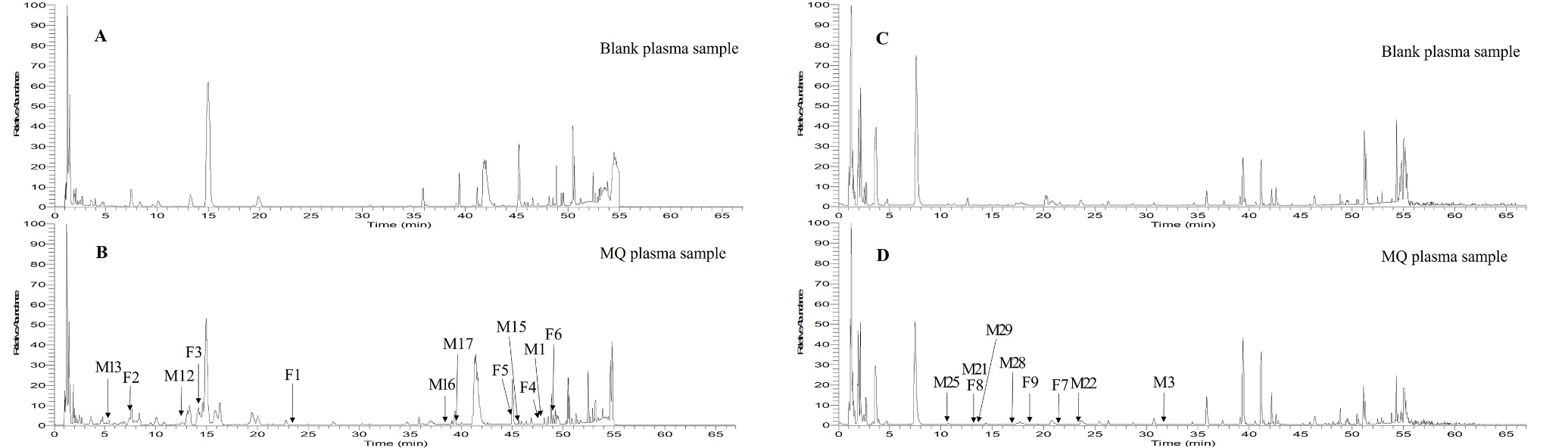


**Supplementary Figure 3.** Base peak chromatograms of absorbed compounds and metabolites in rats’ plasma after administration of *Meconopsis quintuplinervia* extract in negative (A and B) and positive (C and D) ion modes.


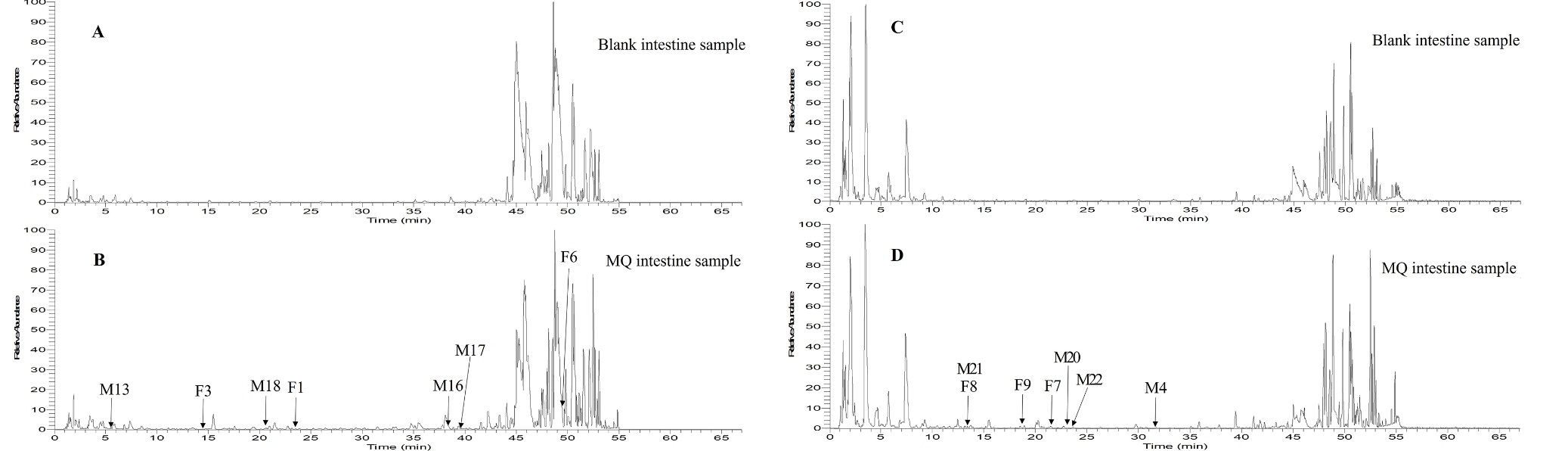


**Supplementary Figure 4.** Base peak chromatograms of absorbed compounds and metabolites in rats’ intestine after administration of *Meconopsis quintuplinervia* extract in negative (A and B) and positive (C and D) ion modes.


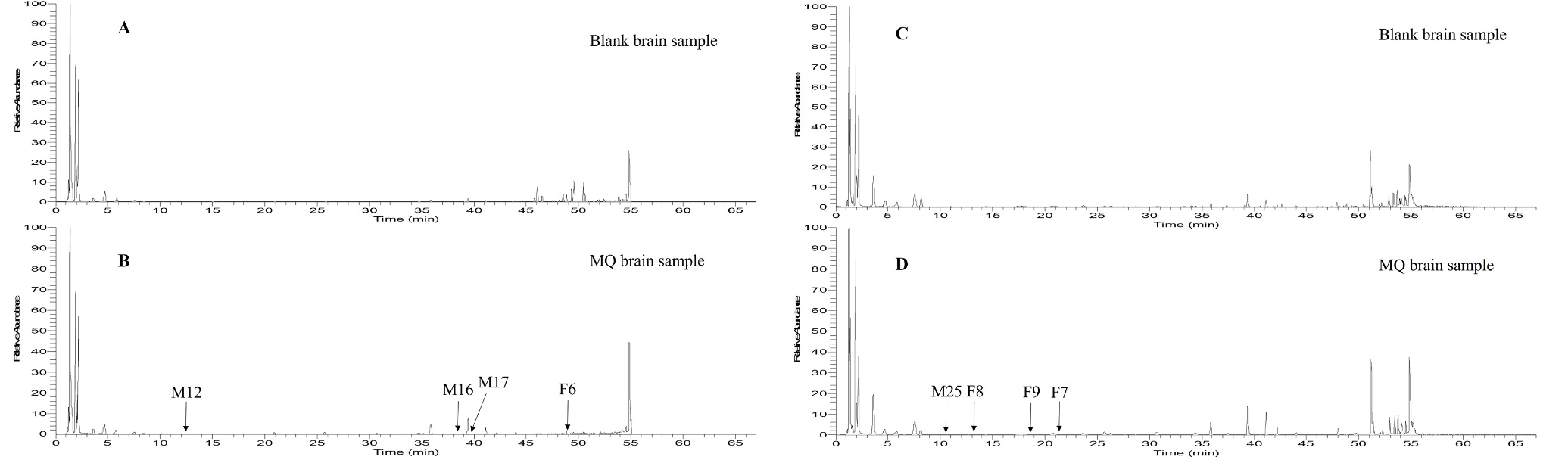


**Supplementary Figure 5.** Base peak chromatograms of absorbed compounds and metabolites in rats’ brain after administration of *Meconopsis quintuplinervia* extract in negative (A and B) and positive (C and D) ion modes.


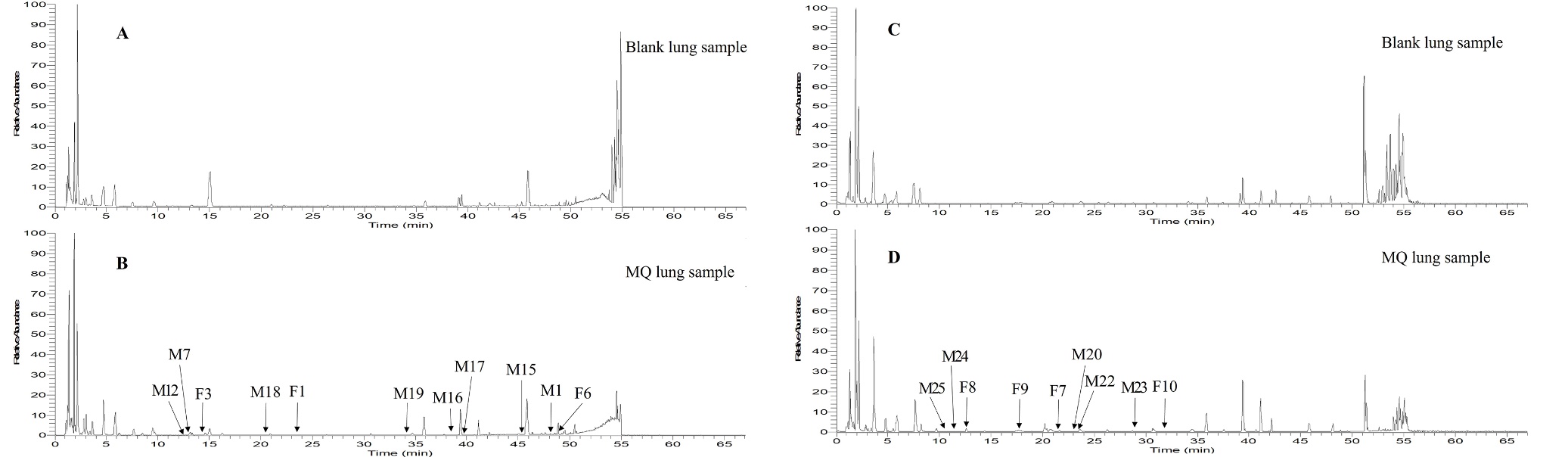


**Supplementary Figure 6.** Base peak chromatograms of absorbed compounds and metabolites in rats’ lung after administration of *Meconopsis quintuplinervia* extract in negative (A and B) and positive (C and D) ion modes.


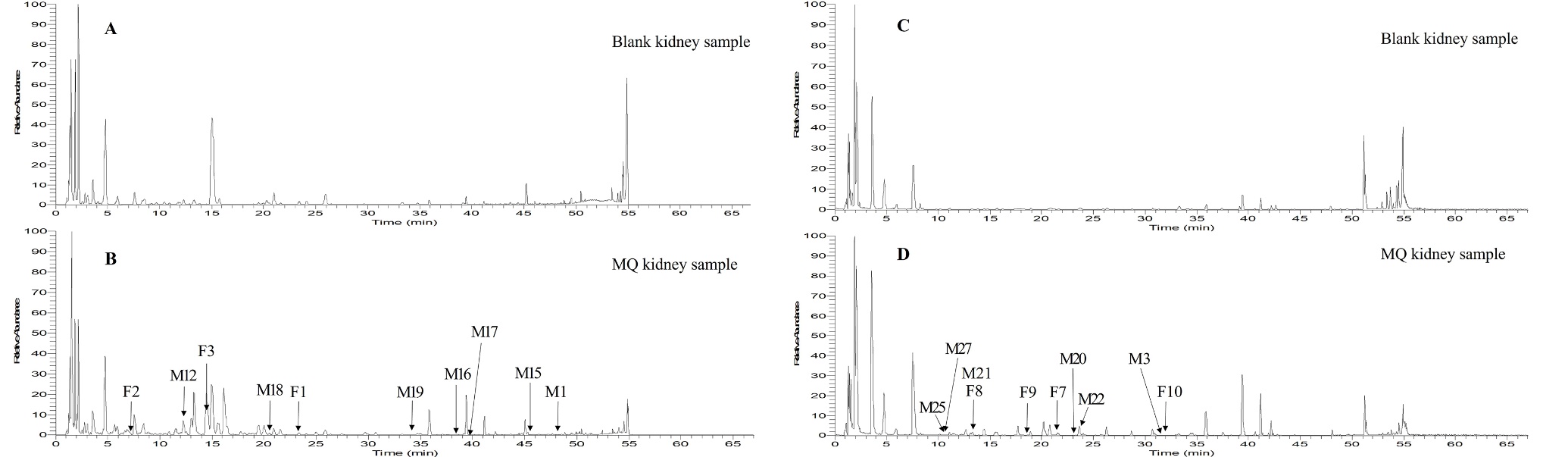


**Supplementary Figure 7.** Base peak chromatograms of absorbed compounds and metabolites in rats’ kidney after administration of *Meconopsis quintuplinervia* extract in negative (A and B) and positive (C and D) ion modes.


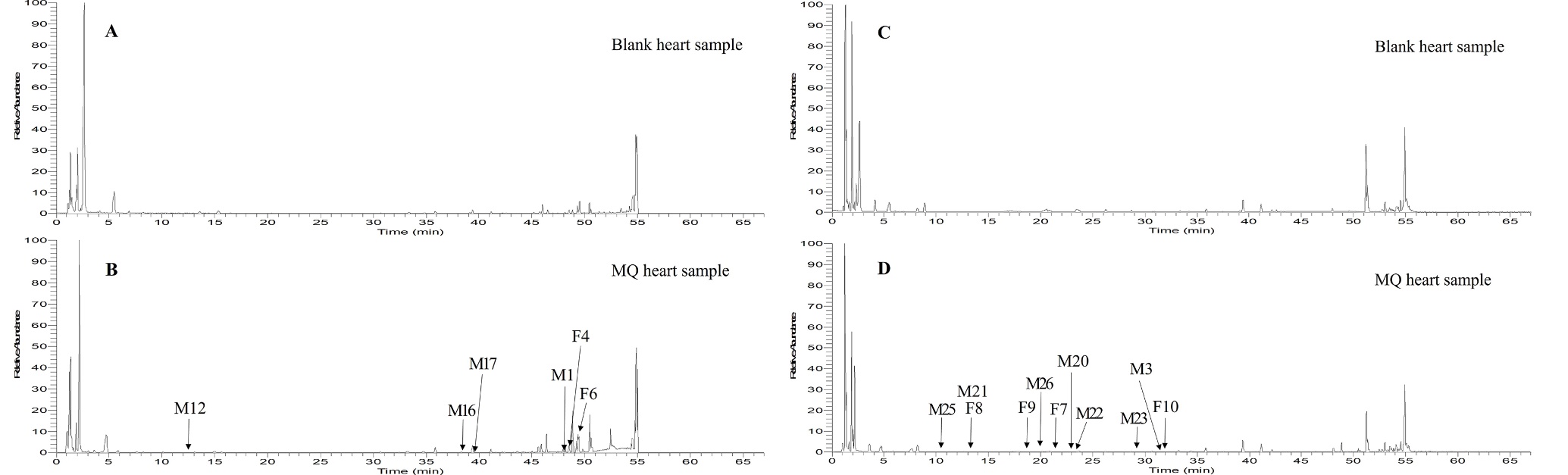


**Supplementary Figure 8.** Base peak chromatograms of absorbed compounds and metabolites in rats’ heart after administration of *Meconopsis quintuplinervia* extract in negative (A and B) and positive (C and D) ion modes.


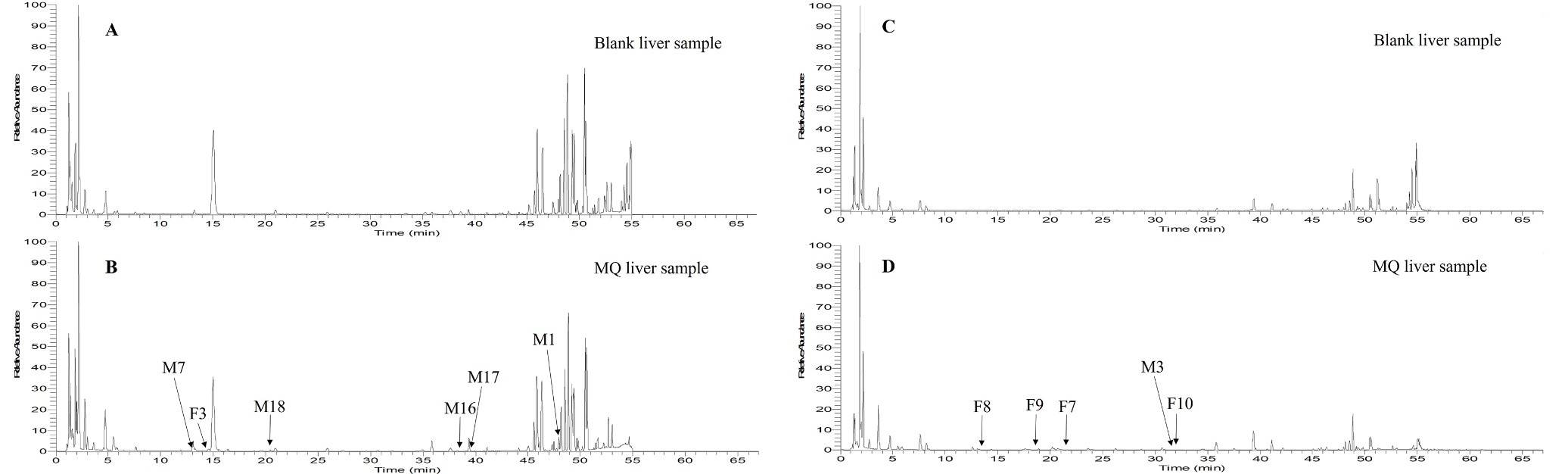


**Supplementary Figure 9.** Base peak chromatograms of absorbed compounds and metabolites in rats’ liver after administration of *Meconopsis quintuplinervia* extract in negative (A and B) and positive (C and D) ion modes.


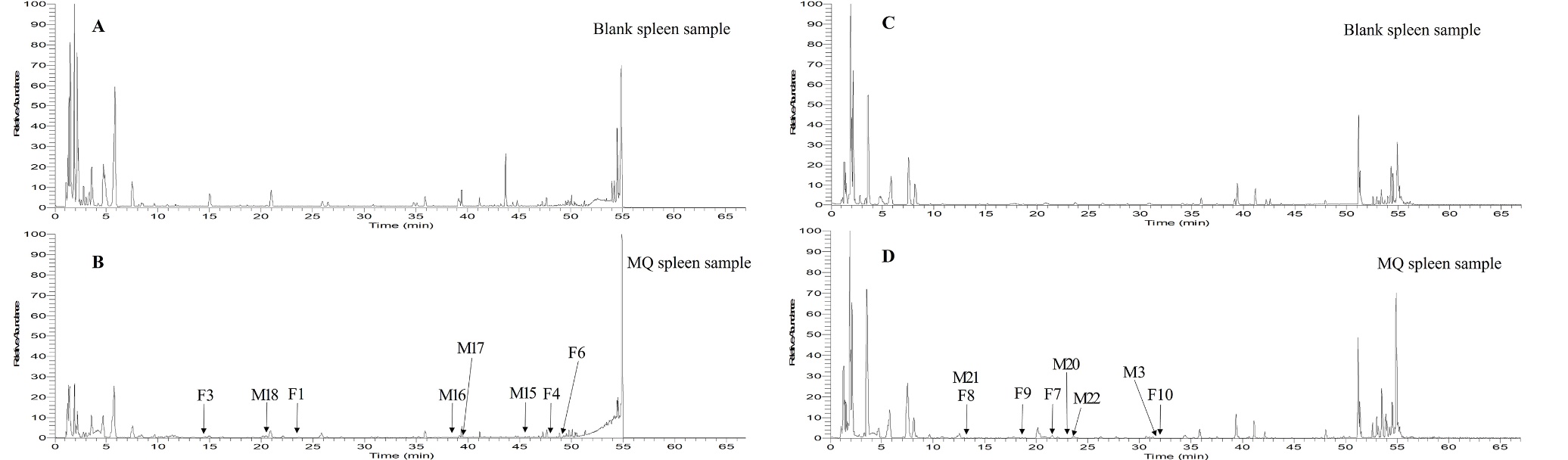


**Supplementary Figure 10.** Base peak chromatograms of absorbed compounds and metabolites in rats’ spleen after administration of *Meconopsis quintuplinervia* extract in negative (A and B) and positive (C and D) ion modes.
